# The Puzzling Fate of a Lupin Chromosome Revealed by Reciprocal Oligo-FISH and BAC-FISH Mapping

**DOI:** 10.3390/genes11121489

**Published:** 2020-12-10

**Authors:** Wojciech Bielski, Michał Książkiewicz, Denisa Šimoníková, Eva Hřibová, Karolina Susek, Barbara Naganowska

**Affiliations:** 1 Department of Genomics, Institute of Plant Genetics, Polish Academy of Sciences, 60-479 Poznan, Poland; mksi@igr.poznan.pl (M.K.); ksus@igr.poznan.pl (K.S.); bnag@igr.poznan.pl (B.N.); 2 Institute of Experimental Botany of the Czech Academy of Sciences, Centre of the Region Hana for Biotechnological and Agricultural Research, 77900 Olomouc, Czech Republic; simonikova@ueb.cas.cz (D.Š.); hribova@ueb.cas.cz (E.H.)

**Keywords:** lupin, FISH, oligo-painting, oligonucleotide probes, comparative-mapping, chromosome evolution, cytogenetics, karyotype evolution, wild species

## Abstract

Old World lupins constitute an interesting model for evolutionary research due to diversity in genome size and chromosome number, indicating evolutionary genome reorganization. It has been hypothesized that the polyploidization event which occurred in the common ancestor of the Fabaceae family was followed by a lineage-specific whole genome triplication (WGT) in the lupin clade, driving chromosome rearrangements. In this study, chromosome-specific markers were used as probes for heterologous fluorescence in situ hybridization (FISH) to identify and characterize structural chromosome changes among the smooth-seeded (*Lupinus angustifolius* L., *Lupinus cryptanthus* Shuttlew., *Lupinus micranthus* Guss.) and rough-seeded (*Lupinus cosentinii* Guss. and *Lupinus pilosus* Murr.) lupin species. Comparative cytogenetic mapping was done using FISH with oligonucleotide probes and previously published chromosome-specific bacterial artificial chromosome (BAC) clones. Oligonucleotide probes were designed to cover both arms of chromosome Lang06 of the *L. angustifolius* reference genome separately. The chromosome was chosen for the in-depth study due to observed structural variability among wild lupin species revealed by BAC-FISH and supplemented by in silico mapping of recently released lupin genome assemblies. The results highlighted changes in synteny within the Lang06 region between the lupin species, including putative translocations, inversions, and/or non-allelic homologous recombination, which would have accompanied the evolution and speciation.

## 1. Introduction

Legumes (Fabaceae Lindl.) are the third largest family of higher plants with approximately 20,000 species, and second as to the harvested area and total production of 300 million metric tons of grain legumes on 190 million ha [1]. The family is diverse in many aspects, including plant morphology, habitat, and ecology, as well as genome size and evolution [2,3]. It has been assumed that the genome complexity and species diversity were promoted by whole-genome duplications (WGDs), which occurred in ancient legumes before the major diversification events [4]. The WGDs were further followed by polyploidization(s) in particular lineages, advancing their further expansions [5,6,7,8,9]. This is a typical evolutionary scenario, which is believed to have occurred in many angiosperm clades. Moreover, the WGDs were found to be related to global climate changes and periods with high diversification rates [10].

Grain legumes are an important source of nutrients for animal feed and human food production. However, the global market has been dominated by one species, a soybean. One of the proposed alternatives to it is the species from the genus *Lupinus* (Fabaceae), which have so far been used as an important component of animal feed (mainly beef and dairy cattle, sheep, pigs, poultry, finfish, and crustaceans) and soil fertilization (based on the symbiosis with nitrogen-fixing bacteria) [11,12]. In Europe, lupins have attracted wide attention in research and innovation programs, highlighted by the incorporation of *Lupinus* crop representatives into numerous European Union initiatives, such as GLIP, LEGATO, LUPICARP, PROTEIN2FOOD, INCREASE, and others. Lupin breeding is most advanced in Australia and many European countries, especially in those located in the eastern part of the continent. Parallel to the growing use of lupins in the food industry, their genomic studies and the knowledge on molecular and evolutionary mechanisms underlying the high variability observed in the genus have been advancing [13,14,15,16,17,18].

Genus *Lupinus* evolved about 50–55 mya and consists of approximately 270 species, subdivided into two groups: Old World lupins (OWLs) and New World lupins (NWLs) [8]. OWLs are native to Mediterranean region and North Africa and consist of 12 annual herbaceous species, including three crops: *L. angustifolius* (Narrow-leafed lupin), *L. albus* L. (white lupin), and *L. luteus* L. (yellow lupin) [19]. More importantly, from the evolutionary point of view, OWLs are characterized by high diversity in genome size (2C DNA amounts ranging from 0.97 pg to 2.44 pg) as well as basic (x = 6–9, 13) and somatic (2*n* = 32–52) chromosome numbers [20,21]. These differences may reflect complex karyotype reorganizations, which occurred during the evolution of this group of plants. Their extant karyotypes were presumably shaped not only by polyploidization, which occurred in the common ancestor of papilionoids, but first of all by whole genome triplication (WGT), which happened at the beginning of the lupine lineage development and were followed by chromosomal rearrangements [13,15,22].

Based on the differences in geographic distribution, morphology (particularly traits of the corolla, pods, and seeds), specific protein polymorphism and alkaloid composition, the OWLs are divided into two groups: smooth-seeded (Malacospermae) group comprising the sections Angustifolius, Albus, Luteus, and Micranthus, and rough-seeded (Scabrispermae) group with the sections Atlanticus and Pilosus [23,24]. Recently identified species *L. mariae-josephi* H. Pascual is similar to Malacospermae in terms of chromosome number, but it is characterized by a unique ‘intermediate’ seed coat structure having common features with both rough- and smooth-seeded species [25]. Despite the recent progress, the knowledge on the course of evolution of species within OWL clade remains limited, and studies addressing evolutionary karyotype changes, especially those involving non-domesticated species, would facilitate the reconstruction of their phylogeny.

Comparative genome analysis can be done by in silico alignment using genome or transcriptome sequences. The use of chromosome-scale scaffolds can provide *a posteriori* insight into the chromosomal changes that took place during the karyotype evolution of the species of interest. Such approach was used recently in *L. albus*, leading to the conclusion that the current karyotype was shaped by 15 fissions and 21 chromosomal fusions, followed by whole genome triplication resulting in 17 major rearrangements [15]. An alternative strategy to in silico sequence-based analysis is the physical localization of specific DNA sequences on chromosomes by fluorescence in situ hybridization (FISH). FISH has been frequently used as a complementary and validation tool for in silico methods, such as fingerprinting-derived contig construction or de novo genome assembly [26]. Soon after its development in the early 1980s, FISH became the most important technique in plant cytogenetics and has been used frequently until now [27]. Indeed, despite so many high-throughput sequencing technologies developed, the number of FISH-based publications in the Web of Science database has not decreased during the past two decades [28].

To date, the most commonly used FISH probes to anchor genome sequences to chromosomes have been bacterial artificial chromosome (BAC) clones as they carry inserts long enough for proper visualization of hybridization signals [26]. In plants with relatively complex genomes, the utility of BAC clones in cross-species comparative studies is limited, mainly due to the abundance of dispersed repetitive sequences in the clones. Thus, probes prepared from unique, single-copy sequences are more useful. However, the limiting factor of the use of single-copy sequences as FISH probes was the scarce availability of repeat-free, unique DNA clones, especially in non-model species with non-sequenced genomes. A significant breakthrough has been achieved recently by the innovations in synthesis and labeling of synthetic oligonucleotides, followed by the release of a publicly available software pipeline (Chorus), which together facilitate the development of locus-specific probes for FISH in a cost-effective manner [28,29]. The identification of large numbers of short (usually 45–50 bp), unique sequences across the whole genome assembly is done by the Chorus software, which enables advanced automation of this process. [29]. Designed oligonucleotides are then massively synthesized, labeled, and divided into pools, ready to use as probes for FISH. The oligo-based approach requires the availability of high-quality genome sequence for the oligonucleotide development, carrying also the representatives of the repetitive fraction of the genome. However, despite the rapid development of DNA sequencing techniques and genome sequence assembly algorithms, genomes of many species are not yet available. A solution may be to design probes using genome assembly from a closely related species, which may allow obtaining ‘universal’ probes that can be used in related species for heterologous hybridization. The ‘oligo-painting’ FISH provides an opportunity for a very precise cross-species analysis of chromosomal rearrangements [28]. The oligo-based technique in the last few years has proven to be effective in karyotyping and chromosome rearrangements identification in *Cucumis*, *Fragaria*, *Solanum,* or *Musa* species [29,30,31,32].

In the present study both traditional (BAC probes) and novel (oligo-painting) FISH approaches were harnessed to provide new insights into karyotype evolution among five OWL species. The species included one domesticated reference *L. angustifolius* (2*n* = 40) and four wild representatives differing in chromosome number, namely *L. cryptanthus* (*2n* = 40), *L. micranthus* (2*n* = 52), *L. cosentinii* (2*n* = 32), and *L. pilosus* (2*n* = 42).

## 2. Materials and Methods

### 2.1. Plant Material

One domesticated and four wild lupin species were used in this study (Table 1). The seeds were provided by the Polish *Lupinus* Gene Bank, Breeding Station Wiatrowo, Poznan Plant Breeders Ltd., Poznań, Poland. These were germinated in Petri dishes at 25 °C to obtain root tips that were suitable for mitotic chromosome isolation. Meiotic pachytene chromosomes were harvested from the young flower buds of the plants cultivated in controlled conditions (16 h of photoperiod, 22 °C; 8 h of night, 18 °C) in the Plant Growing Center of the Institute of Plant Genetics, Polish Academy of Sciences.

### 2.2. BAC Clone DNA Isolation and Labeling

Single copy BAC clones from the *L. angustifolius* nuclear genome BAC library [33] identified as Lang06-specific in the previous study [17] were used. Due to the dispersed mapping pattern of 067H16 BAC clone in wild lupins, one additional probe (059F07) specific to Lang06 [34] was used instead. Moreover, BAC clone 127N17 was also not included in FISH because of its overlapping sequence with the BAC 051D03. The complete list of used BAC clones and their alignment to pseudochromosomes is included in the Supplementary Materials (Supplementary Table S1). DNA isolation from BAC clones was performed using miniprep kits (QIAprep Spin; Qiagen, Hilden, Germany). BAC DNA thus obtained was labeled by nick-translation (Sigma–Aldrich, St. Louis, MI, USA), using either digoxigenin-11-dUTP (Sigma–Aldrich) or tetramethylrhodamine-5-dUTP (Sigma–Aldrich). BAC clone DNA isolation and labeling were done as described by Susek et al. [35]. To obtain information on localization of BAC clones in the genome assembly, nucleotide sequences of inserts were downloaded from the NCBI database (accession numbers provided in Table S1) and aligned to the *L. angustifolius* pseudomolecules and/or scaffolds [18] using Basic Local Alignment Search Tool (BLAST) implemented in Geneious 9.1.8 program (Biomatters, Ltd., Auckland, New Zealand).

### 2.3. Oligonucleotide Probe Design, Synthesis, and Labeling

**Figure 1:**
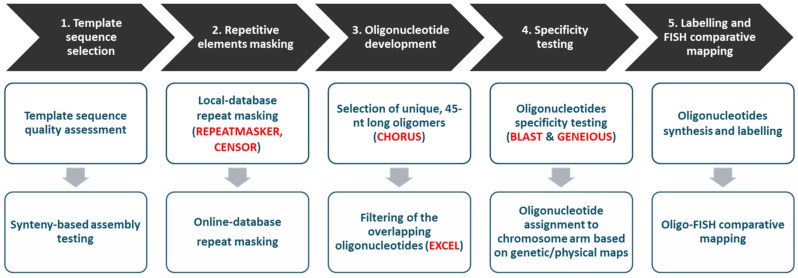
General scheme of oligonucleotide-based probe development.

The procedure for preparing the oligonucleotide probes consisted of several successive steps shown in Figure 1.

Lang06 pseudochromosome (accession number CP023118) from the reference genome sequence of *L. angustifolius* cv. Tanjil [18] was selected. The total Lang06 pseudochromosome sequence length was 40,902,325 nt. The analysis of the Lang06 pseudochromosome included BLAST alignments to the sequences of related species: *L. albus* transcriptome aligned with *L. albus* genetic map [36], *Arachis duranensis* and *A. ipaensis* genomic sequences [37], and an earlier version of the *L. angustifolius* genome [38]. Based on the Megablast algorithm (word size: 28, e-value: 1 × 10^−10^) performed in Geneious 9.1.8, no candidate miss-assemblies (>100 bp) were detected in the Lang06 pseudochromosome. Repetitive elements were masked subsequently using RepeatMasker [39] and CENSOR programs [40]. Chorus software [29] (Madison, WI, USA; github.com/zhangtaolab/Chorus2/) was used to generate a set of unique, 45 nt oligomers with default parameters (75% homology, dTM 10) based on the template sequence with masked repeats. Obtained oligonucleotides were mapped on the reference *L. angustifolius* cv. Tanjil sequence in Geneious 9.1.8, to determine their location in the genome (pseudochromosome) and the number of expected binding sites. This analysis was supplemented by the specificity test using BLAST with gradually decreasing similarity (to about 75%) and re-mapping of the oligonucleotides. Assignment of developed probes to the particular arms of the Lang06 chromosome was carried out by comparison of the density of markers on the *L. angustifolius* genetic map [41] with the physical distance between these markers. A significant drop in marker density combined with an increase in physical distance was interpreted as (peri)centromere. As lupin centromere regions are composed of many simple sequence repeats, oligonucleotides localized around centromeres were discarded from the probe synthesis.

Four libraries, each comprising 8000–20,000 oligonucleotides (45-mers), were synthesized by Arbor Biosciences (Ann Arbor, MI, USA). For the initial two libraries (O1 and O2), unlabeled ‘immortal’ oligonucleotides were ordered, and the probe labeling was performed according to Han et al. [29]. Briefly, the libraries were amplified using emulsion PCR [42], with F primer containing T7 RNA polymerase promoter, then washed with water-saturated diethyl ether and ethyl acetate, followed by a purification step using a QIAquick PCR purification kit (Qiagen). Obtained DNA (~480 ng) was subjected to T7 in vitro transcription using a MEGAshortscript T7 Kit (ThermoFisher Scientific/Invitrogen, Waltham, MA, USA) at 37 °C for 4 h. The next step involved purification of the RNA using RNeasy Mini Kit (Qiagen) and reverse-transcription with either digoxigenin- or biotin-labeled R primer (Eurofins Genomics, Ebersberg, Germany) using Superscript II Reverse Transcriptase and SUPERase-In RNase inhibitor (ThermoFisher Scientific/Invitrogen). Finally, the RNA:DNA hybrids were cleaned with Quick-RNA MiniPrep Kit (Zymo Research, Freiburg im Breisgau, Germany) and hydrolyzed with RNase H (New England Biolabs, Ipswich, MA, USA) and RNase A (ThermoFisher Scientific/Invitrogen). To obtain single-stranded labeled oligonucleotide-probes, the additional purification with a Quick-RNA MiniPrep Kit (Zymo Research) was performed, followed by nuclease-free water elution. The remaining two libraries (O3 and O4) were synthetized by Arbor Biosciences as ready-to-use FISH probes, labeled with either digoxigenin or biotin.

### 2.4. Fluorescence In Situ Hybridization

Preparation of mitotic metaphase spreads and FISH with BAC-based probes (BAC-FISH) was done according to Susek et al. [17] with minor modifications. Young roots (~1 cm long) were treated in a solution of 40% (*v*/*v*) pectinase (Sigma–Aldrich, Darmstadt, Germany), 3% (*w*/*v*) cellulase (Sigma–Aldrich), and 1.5% (*w*/*v*) cellulase ‘Onozuka R-10′ (Serva, Heidelberg, Germany), in 37 °C for 60 min (lateral roots) or 100 min (primary roots).

Meiotic chromosome preparations were made from anthers collected from young buds. The buds were harvested and fixed in a solution of 96% ethyl alcohol/glacial acetic acid in a ratio of 3:1. The solution was not changed to a new one until the buds were completely discolored and then stored at −20 °C. The fixative was removed by a series of rinses in water and in citrate buffer. Single anthers or small buds devoid of crown petals were isolated using a stereoscopic microscope (SZX7 Olympus, Shinjuku, Tokyo, Japan). The material was digested using an enzyme cocktail, including 10% (*v*/*v*) pectinase (Sigma-Aldrich), 0.1% (*w*/*v*) cellulase (Sigma–Aldrich), and 0.1% (*w*/*v*) cytohelicase (Sigma–Aldrich) and incubated at 37 °C for about 150 min. Then the material was suspended in citrate buffer at 4 °C for 30 min. Finally, individual anthers were suspended in a drop of 60% acetic acid and isolated/transferred to a degreased glass slide. The material was covered with a coverslip and gently squashed. The quality of the material (number of meiotic divisions, degree of chromosome condensation, presence of cytoplasm) was assessed under the phase contrast microscope (BX41/CX41 Olympus). Selected high-quality slides were frozen at −80 °C (or on dry ice), the coverslip was removed and then dehydrated in 99.8% ethyl alcohol cooled to −20 °C for 30 min and dried at room temperature. The final quality was assessed under a phase contrast microscope, and then the slides were stored at −20 °C until use.

To localize the signals in both mitotic and meiotic stage, the chromosomes were counterstained with 4′,6-diamidino-2-phenylindole (DAPI) in Vectashield Antifade Mounting Medium (Vector Laboratories, Burlingame, CA, USA). The fluorescent signals were acquired and examined using F-View monochromatic camera attached to an Olympus BX-60 epifluorescence microscope, pseudocolored in Wasabi (Hamamatsu Photonics, Hamamatsu, Shizuoka, Japan), superimposed using Micrografx (Corel Corporation, Ottawa, ON, Canada) Picture Publisher 10 software and GIMP 2.8.20.

Oligo-FISH procedure was as follows: selected slides with meiotic chromosomes were washed in 4% formaldehyde in 2 × Saline Sodium Citrate (SSC) buffer at room temperature for 10 min, then dehydrated in ethanol series for 2 min each (70%, 90%, and 99.8%). Then, the hybridization mix containing 50% (*v*/*v*) formamide, 10% (*w*/*v*) dextran sulfate in 2 × SSC, and 10 ng/μL of the labeled probe was added onto a slide and denatured at 80 °C for 3 min. Hybridization was carried out overnight at 37 °C. The particular probes (labeled with digoxigenin and biotin) were detected using anti-digoxigenin-FITC (Roche Applied Science, Penzberg, Germany) and streptavidin-Cy3 (ThermoFisher Scientific/Invitrogen), respectively. The chromosomes were counterstained with DAPI in Vectashield Antifade Mounting Medium (Vector Laboratories). Fluorescent signals were acquired and examined with Axio Imager Z.2 Zeiss microscope (Zeiss, Oberkochen, Germany) with Cool Cube 1 camera (Metasystems, Altlussheim, Germany). The capture of fluorescence signals and merging the layers were performed with ISIS software 5.4.7 (Metasystems, Heidelberg, Germany). Alternatively, the signals were acquired and examined with the hardware and software as described for BAC-FISH.

## 3. Results

### Oligonucleotide-Based Probe Development and Oligo-FISH

When selecting a suitable template for the oligonucleotide probes, the following requirements were considered:

- The chromosome region should exhibit at least partial differentiation in related species, evidenced by previous cytogenetic studies or genome/linkage mapping;
- The chromosome region should have a low abundance of repetitive elements to allow the design of unique probes;
- Scaffolding in this region should be strongly supported by linkage mapping to avoid unintentional incorporation of fragments from other chromosomes;
- Chromosome-specific cytogenetic landmarks (i.e., BAC clones) should be available for this region to enable parallel use of two techniques—BAC-FISH and oligo-FISH.

Considering BAC-FISH results obtained in previous studies [17,35] and comparative mapping of *L. angustifolius* and *L. albus* genome assemblies and linkage maps [18,36,37], the pseudochromosome Lang06 sequence of *L. angustifolius* cv. Tanjil was selected as a template for oligonucleotide design. The first set of oligonucleotides was divided into two pools (libraries), specific for both arms (A and B) of the Lang06 chromosome. Based on the results of oligo-FISH, two more pools were later selected from the arm B of Lang06 to allow for fine mapping. The complete list of selected oligonucleotides is available at doi.org/10.5281/zenodo.4226537. A detailed description of each library is shown in Table 2.

**Figure 2:**
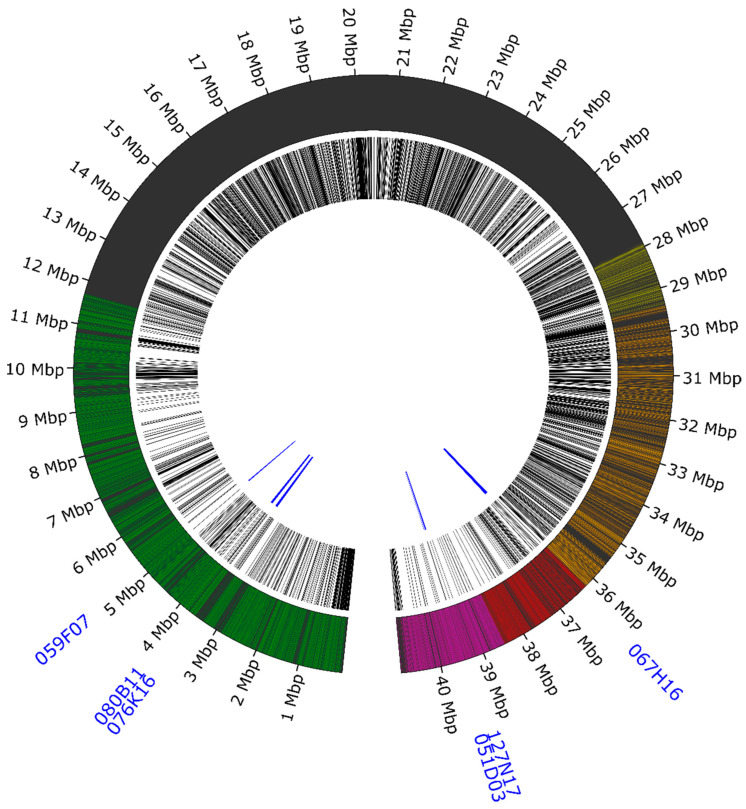
Schematic layout of the oligonucleotide libraries and repetitive sequences in pseudochromosome Lang06. The O1 library is marked in green, the O2 library in red, and the O3 library in yellow. Orange highlights the common region for the O2 and O3 libraries, whereas violet covers the region common to O2 and O4 libraries. Repetitive sequences are shown in black in the middle circle. Bacterial artificial chromosome (BAC) clone localization diagram is shown in blue in the inner circle.

The combined scheme of the chromosomal distribution of the developed oligonucleotide libraries, including localization of the used Lang06-specific BAC clones and the detected repetitive elements, is shown in Figure 2.

**Figure 3:**
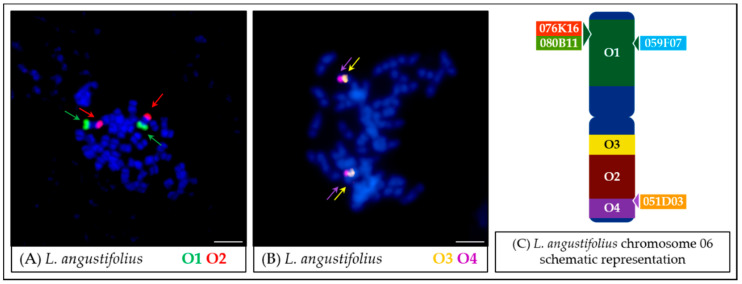
Fluorescence in situ hybridization (FISH) mapping of oligonucleotide probes in mitotic chromosomes of *L. angustifolius*. The positions of individual probes are marked by arrows. Probe colors are as follows: green (O1, Lang06 arm (**A**), red (O2, Lang06 arm (**B**)), yellow (O3, pericentromeric region of Lang06 arm B) and purple (O4, telomere region of Lang06 arm B). Scale bar: 5 μm. Chromosome Lang06 schematic representation (**C**), showing the positions of aligned particular oligonucleotide-based or BAC-based probes, was not drawn to scale.

The ready-to-use labeled oligonucleotide probes were hybridized to mitotic metaphase chromosome spreads of *L. angustifolius* cv. Tanjil, which served as a reference species and the template for the 45-nt oligomer development. Oligo-FISH resulted in visible signals covering chromosome Lang06 arms (Figure 3A,B). The Oligo1 (O1) probe mapped specifically to the A arm, the Oligo2 (O2) probe to the B arm (Figure 3A), the Oligo3 probe (O3) hybridized to the pericentromeric region of the B arm, while the Oligo4 (O4) probe with the telomere region of the B arm (Figure 3B). These observations confirmed that the probes hybridize specifically to target regions in the *L. angustifolius* genome. Moreover, the presence of locus-specific signals (i.e., the lack of dispersed signals) provided evidence that the repetitive sequence filtering process, based on several rounds of RepeatMasker and CENSOR masking, was effective. The localizations of all probes were also confirmed in meiotic chromosomes by two consecutive oligo-FISH reactions performed on the same slide. Images of oligo-FISH on *L. angustifolius* meiotic sample are presented in Supplementary Figure S1.

**Figure 4:**
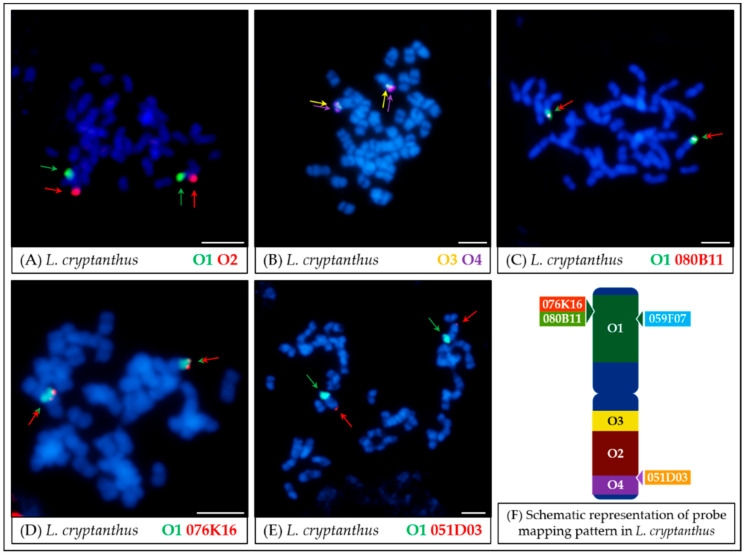
FISH mapping of oligonucleotide probes (**A**,**B**) and oligonucleotide combined with BAC clones (**C**–**E**) on mitotic metaphase chromosomes of *L. cryptanthus*. The positions of individual probes are marked by arrows. Probe colors are as follows: green (O1, Lang06 arm A), red (O2, Lang06 arm B), yellow (O3, pericentromeric region of Lang06 arm B) and purple (O4, telomere region of Lang06 arm B). Scale bar: 5 μm. Schematic representation of probe mapping pattern in *L. cryptanthus* chromosomes (**F**), showing observed positions of particular oligonucleotide-based or BAC-based probes, was not drawn to scale.

The hybridization pattern of individual oligonucleotide probes in *L. cryptanthus* chromosomes remained identical to the reference species *L. angustifolius*: the O1 probe mapped in the A arm of the Lcry06 chromosome (Figure 4A), while the remaining probes hybridized to the Lcry06 arm B (Figure 4B). The results were verified by comparative mapping of the O1 probe with the Lang06-specific BAC clones: 080B11 (Figure 4C), 076K16 (Figure 4D), 051D03 (Figure 4E), and 059F07. The series of attempts was made to precisely visualize the oligonucleotide probes on meiotic material in studied wild lupin species, but the low signal intensity rendered the attempts unsuccessful.

**Figure 5:**
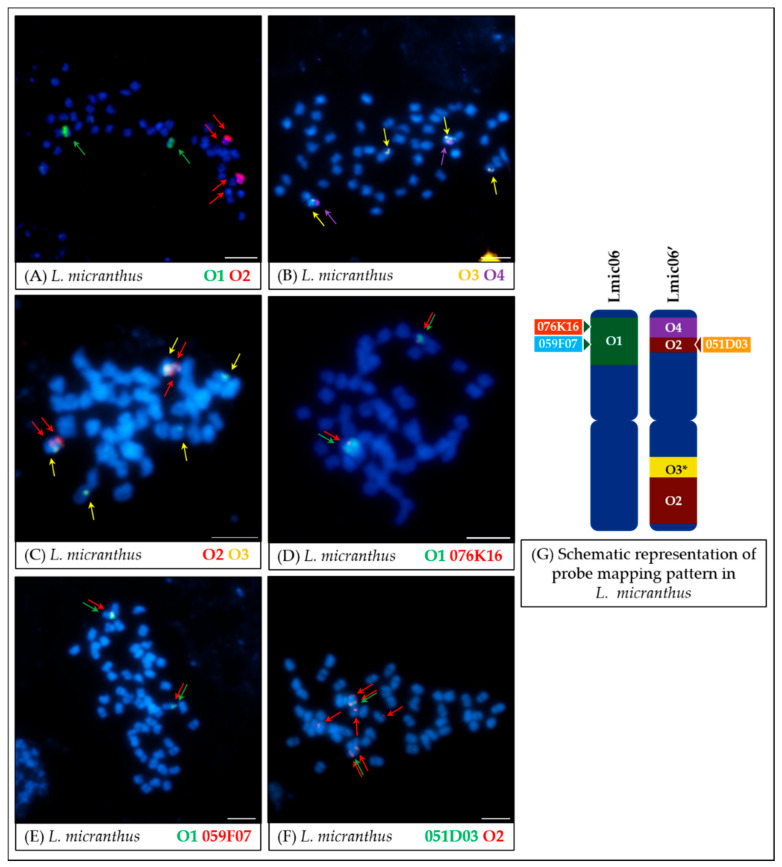
FISH mapping of oligonucleotide probes (**A**–**C**) and oligonucleotide probes combined with BAC clones (**D**–**F**) on mitotic chromosomes of *L. micranthus*. The positions of individual probes are marked by arrows. Probe colors are as follows: green (O1, Lang06 arm A), red (O2, Lang06 arm B), yellow (O3, pericentromeric region of Lang06 arm B), and purple (O4, telomere region of Lang06 arm B). Scale bar: 5 μm. Schematic representation of probe mapping pattern in *L. micranthus* chromosomes (**G**), showing observed positions of particular oligonucleotide-based or BAC-based probes, was not drawn to scale. O3*—beside two major loci, minor signals were also noticed.

Comparative cytogenetic mapping in *L. micranthus* chromosomes showed that the structure of the arm A of the chromosome Lmic06 remained unchanged compared to *L. angustifolius* (Figure 5A). It was also revealed that the O1 probe co-localized with BAC clones 076K16, 059F07 (Figure 5D,E), and 080B11 (not shown). Structural differences in *L. micranthus* were revealed using probes from the arm B of the Lang06 chromosome; namely, the O2 probe was mapped to both arms of the chromosome. Because of the difference from Lmic06, it is named here as Lmic06′ (Figure 5A). BAC clone 051D03 detected on the B arm of Lang06 was co-localized with O2 on the A arm of Lmic06′ (Figure 5F). The O3 and O4 probes split into two separate arms of this Lmic06′ chromosome (Figure 5B,C). In case of the O3 probe, beside the two major loci, minor weaker signals were also observed in the chromosomes other than Lmic06 or Lmic06′ (Figure 5B,C).

**Figure 6:**
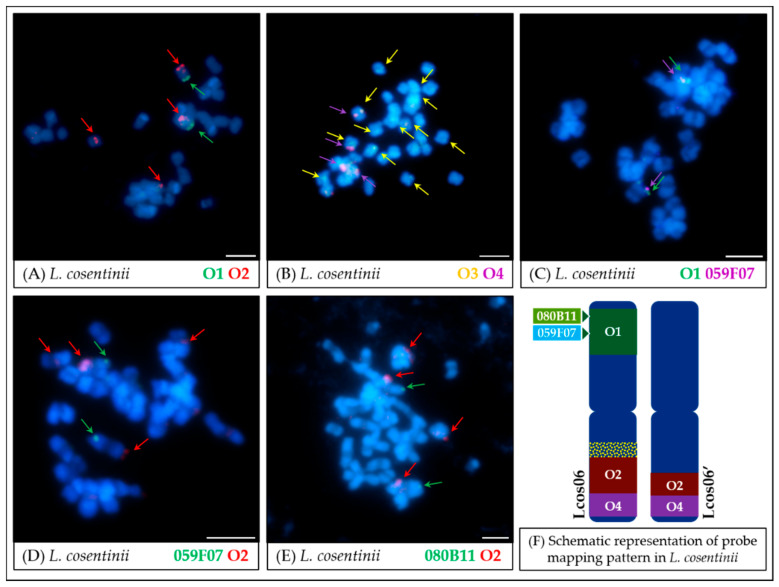
FISH mapping results of oligonucleotide probes (**A**,**B**) and oligonucleotide combined with BAC clones (**C**–**E**) in mitotic chromosomes of *L. cosentinii.* The positions of individual probes are marked by arrows. Probe colors are as follows: green (O1, Lang06 arm A), red (O2, Lang06 arm B), yellow (O3, pericentromeric region of Lang06 arm B), and purple (O4, telomere region of Lang06 arm B). Scale bar: 5 μm. Schematic representation of probe mapping pattern in *L. cosentinii* chromosomes (**F**), showing observed positions of particular oligonucleotide-based or BAC-based probes, was not drawn to scale. The O3 probe hybridized to multiple loci.

The intensity of oligo-FISH signals was noticeably weaker in *L. cosentinii*. The specificity of the O1 probe to the arm A of the Lcos06 chromosome was preserved, whereas both O2 and O4 probes hybridized to two loci, the first localized in the arm B of the same chromosome as the O1 probe (Lcos06) and the second in a different chromosome (Lcos06′) (Figure 6A,B). The O3 probe revealed signals dispersed over multiple loci on *L. cosentinii* chromosomes. To confirm the results of oligo-FISH, combinations of selected BAC clones with oligonucleotide probes were analyzed. These experiments showed that clone 059F07 shared the locus with the O1 probe (Figure 6C). Both 059F07 and 080B11 shared the chromosome (Lcos06) with the O2 probe (Figure 6D,E). Other BAC clones (051D03, 076K16) hybridized to multiple loci.

**Figure 7:**
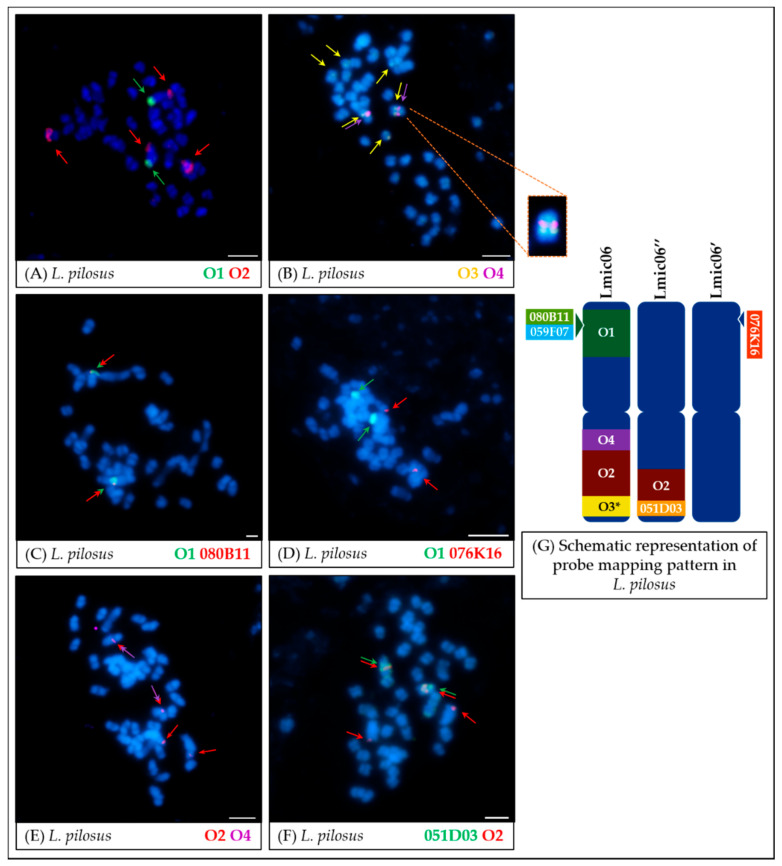
FISH mapping of oligonucleotide probes (**A**,**B**) and oligonucleotide probes combined with BAC clones (**C**–**F**) on mitotic chromosomes of *L. pilosus*. The positions of individual probes are marked by arrows. Probe colors are as follows: green (O1, Lang06 arm A), red (O2, Lang06 arm B), yellow (O3, pericentromeric region of Lang06 arm B), and purple (O4, telomere region of Lang06 arm B). Scale bar: 5 μm. Schematic representation of probe mapping pattern in *L. pilosus* chromosomes (**G**), showing observed positions of particular oligonucleotide-based or BAC-based probes, was not drawn to scale. O3*—besides two major loci, minor signals were also noticed. The fragment of Figure 7B showing the intra-chromosomal inversion was magnified (marked with orange, dashed line).

In the last of the analyzed species, *L. pilosus*, a unique karyotyping pattern was observed. The O1 probe hybridized to the arm A of the chromosome Lpil06 and co-localized with BAC clone 080B11 (Figure 7A,C) and 059F07 as in the reference species, while the BAC clone 076K16 mapped to a different chromosome (Lpil06′, Figure 7D). Although the remaining probes (O2, O3, and O4) hybridized to the arm B of the Lpil06 chromosome (as in reference species), their chromosome arrangement was reversed compared to the *L. angustifolius*: O3 hybridized to the near-telomere region, whereas O4 in the pericentromeric region (see enlarged fragment of Figure 7B). Moreover, beside the two major loci, minor weaker signals in other chromosomes were noticed during the O3 probe mapping (Figure 7B). Interestingly, the O2 probe hybridized to two separate chromosomes (Lpil06 and Lpil06′’ in which the O2 probe was co-localized with BAC clone 051D03, Figure 7F), and both of these were different from the chromosome Lpil06′ on which BAC 076K16 was mapped (Figure 7D). Noteworthy, BAC clone 051D03 hybridizing to a different chromosome than Lpil06 was confirmed in our previous research using FISH comparative mapping with BAC clone 080B11 [17].

## 4. Discussion

### 4.1. Development of Oligonucleotide Probe Sets

The BAC-FISH method provided preliminary insight into the diversification of karyotype structure among the studied lupin species [17,35]. Due to the fact that BAC clones covered only short fragments of chromosomes, there was a need to develop a new type of probe applicable for comparative mapping and covering substantially larger genomic regions, including the entire chromosome arms. Such an approach would allow for a more detailed examination of the differences between species. One of the possible solutions is a massive synthesis of oligonucleotide probes covering the unique regions of the chromosome [43]. As recently demonstrated in *Cucumis* [29], such probes can be used in heterologous FISH to visualize structural chromosomal differences between species that differentiated up to 12 Mya. However, a lower intensity of fluorescent signals should be taken into account.

Whole-genome triplication, which is believed to predate *Lupinus* lineage evolution, occurred about 24.6 Mya [38], whereas differentiation of particular OWL species has been dated from about 1 to 10 mya [8,13,44]. Given these estimations, the evolutionary age of closely related nodes in OWL clade fits within the suggested limitations for the use of heterologous oligo-FISH. The optimal approach for OWL comparative mapping would comprise the hybridization of oligonucleotide probes designed for all *L. angustifolius* chromosomes. Such a strategy was implemented in *Zea mays* L. [45], when sets of oligonucleotide probes were developed, each of them designed to bind specifically to a single chromosome, making it possible to identify all 10 chromosomes of this species in consecutive FISH reactions. The undoubted constraint of such an approach when used in species with higher number of chromosomes would be linear multiplication of costs associated with the synthesis of chromosome-specific oligonucleotide sets. Moreover, such studies in OWLs are currently hampered by the uncertainties in super scaffold assembly constituting one of the reference *L. angustifolius* genome versions, highlighted by differences between the two recently published versions of the sequence [18,38]. Therefore, according to the assumption that the high quality of the template directly translates into the quality of the designed probes [28,29], Lang06 sequence was preselected for oligonucleotide probe design in this work. Our in silico comparative analysis indicated that this pseudochromosome does not contain significant errors (missing or incorrect fragments from other chromosomes), which could hinder the specificity of the developed probes. Moreover, our previous BAC-based studies highlighted this chromosome as a good candidate to track large-scale rearrangements [17]. Indeed, when *L. angustifolius* and *L. albus* genome assemblies were aligned to each other, Lang06 was found to be split between Chr16 and Chr23 in the latter species [13,15].

The oligonucleotide probes designed in this study were characterized by parameters similar to those used in the studies of *Cucumis* and *Solanum* species [29,31], namely the length (45 nt), homology (>75%), and the difference between the probes and hairpins Tm (dTM 10). Oligo–FISH performed on the mitotic and meiotic *L. angustifolius* chromosomes showed that all four probes mapped specifically in the Lang06 chromosome (Figure 3), consistent with the assumptions made during the probe design (Figure 2). The high specificity of developed probes highlighted the correctness of the Lang06 pseudochromosome assembly, at least in the scaffolds covered by the probes. However, it should be noted that some potential discrepancies (such as the incorrect orientation of sequence fragments covered by individual probes as well as the absence or multiplication of specific regions of the sequence) could go unnoticed due to the established parameters of the FISH reaction (stringency) and the characteristic of the designed probes. Thus, oligonucleotides labeled with a common fluorescent label are the source of a uniform signal, regardless of their arrangement within a single pool. The absence of fragments up to 1 Mbp in the template sequence may also go unnoticed due to the resolution limitations of the FISH performed on mitotic metaphase chromosomes [46]. This issue can be partially resolved by analyzing the fluorescence signal in a less condensed chromatin stage. To exemplify, such an oligo-FISH approach performed on *Musa acuminata* chromosomes revealed minor discrepancies in genomic sequence orientation in the form of the inversion of arms of the 1st, 6th, and 7th chromosomes in relation to the karyotype [32]. It is also worth emphasizing that, in our study, the mapping pattern of the developed probes in Lang06 reflected their organization in the pseudochromosome sequence in the libraries specific for individual arms (sets of oligonucleotide probes O1 and O2), as well as in the opposite regions of the B Lang06 arm (O3 and O4).

### 4.2. Comparative Mapping of Wild Lupin Species Using Oligonucleotide Probes

The Oligo–FISH results in *L. cryptanthus* (Figure 4) were identical to those obtained for the reference species, including the mapping pattern of oligonucleotide probes together with BAC clones. Each of the four oligonucleotide probes specifically mapped to a single region of the Lcry06 chromosome, and the intensity of hybridization signals was higher as compared to other species (*L. micranthus*, *L. cosentinii,* and *L. pilosus*). This was consistent with the previous studies involving BAC-based probes [17,35] and in line with the hypothesis that *L. cryptanthus* is a wild form of *L. angustifolius* [21]. Genomic sequences of Lang06 and Lcry06 chromosomes seem very similar because there were no significant (and observable with the methods used) changes in the regions marked by oligonucleotide probes.

On the other hand, in *L. micranthus*, the oligo-FISH analysis showed the existence of significant structural genomic differences between this species and *L. angustifolius* (Figure 5). Similar to the BAC–FISH results [17], probes from different arms of the chromosome Lang06 landed onto two separate *L. micranthus* chromosomes (Lmic06 and Lmic06′). The novel information provided by the oligonucleotide-based approach was that the O2 probe mapped to both arms of the Lmic06′, on the A arm co-localizing with both the O4 and BAC clone 051D03, and on the B arm with the O3 probe. Moreover, weaker signals, which were noticed during O3 probe mapping, might be related to the propagation of repetitive elements or duplication/insertion of short sequence fragments in the *L. micranthus* genome, collinear to the pericentromeric regions of Lang06. It should be noted that to visualize both 051D03 and O2 probe in FISH, the stringency was lowered (to about 65%); hence additional signals were visible for O2 (Figure 5F).

The analysis of the hybridization pattern of the oligonucleotide probes in *L. cosentinii* in reference to *L. angustifolius* (Figure 3 and Figure 6) revealed that only one probe from the Lang06 arm A mapped to a single locus, retaining the reference pattern. Two probes from the B arm (O2 and O4) were mapped together in the B arm of Lcos06 chromosome, but also hybridized to another chromosome (Lcos06′). This might be the result of duplication and/or translocation of the arm B of Lang06 chromosome (containing O1 and O2 probes) to Lcos06′ chromosome. The O3 probe in *L. cosentinii* was the only oligonucleotide probe that hybridized to multiple loci. It is probably a reflection of a specific type(s) of repetitive sequences in the *L. cosentinii* genome, contributing to the ‘dispersed’ mapping pattern of the O3 probe (and BAC clones 051D03 and 076K16). It is possible that some of the oligonucleotides in the pericentromeric region of *L. angustifolius* (which the O3 probe was designed to target) belong to the group of repetitive sequences represented more abundantly (or grouped in appropriate clusters) in the genome of *L. cosentinii* than in the reference species. The presence of specific repetitive elements may also explain weak, additional signals observed during O3 probe mapping in *L. micranthus* and *L. pilosus* chromosomes.

The most recently diversified [8] among the analyzed set of species is *L. pilosus*, which generally revealed a similar Oligo–FISH mapping pattern to the reference *L. angustifolius* (Figure 7). However, the segment order in the arm B of Lpil06 chromosome was reversed as compared to the Lang06, most likely due to paracentric inversion. It was also noted that the O2 probe hybridized to loci on two chromosomes (Lpil06 and Lpil06′), highlighting the remnants of a hypothetical translocation/duplication. O1 probe in *L. pilosus* retained its reference hybridization pattern contrary to the BAC clone 076K16 from the same arm, which was mapped on a different chromosome. Sequence alignment of 076K16 clone to the *L. angustifolius* genome with anchored oligonucleotides showed a significant (>30%) decrease in the frequency of oligonucleotides comprising the O1 probe in the region matching BAC clone sequence, as compared to the average oligonucleotide density of the O1 probe. Most likely, this decrease was due to the specific sequence properties in this genome region (e.g., the presence of palindromes or low complexity sequences) that disrupted the design of short oligonucleotide probes but not necessarily interfering with the hybridization of longer sequences, such as BAC clones. Noteworthy, alignment of 076K16 clone sequence to the *L. albus* genome assembly [15] resulted in three distinct, high-score matches (hits on chromosomes Lalb05, Lalb09, and Lalb16) with numerous rearrangements, which implies complex evolutionary reshuffling (Supplementary Table S2). The decrease in the frequency of oligonucleotides, along with potential evolutionary sequence changes, may explain the fact that no signal was detected at the same locus when the O1 probe and BAC clone 076K16 were mapped simultaneously in *L. pilosus* (Figure 7). On the other hand, the difference in loci between the BAC clone and the O1 probe itself may indicate the translocation of a short sequence fragment (including this clone) to another chromosome. Such a phenomenon has been observed, among others, in *L. angustifolius* for the *LanFTc2* gene, which is located in the Lang17 chromosome region with a very high degree of collinearity within legumes. However, none of these collinear regions contains a corresponding homolog of this gene, despite the presence of such homologs for other genes adjacent to *LanFTc2* [47,48].

## 5. Conclusions

**Figure 8:**
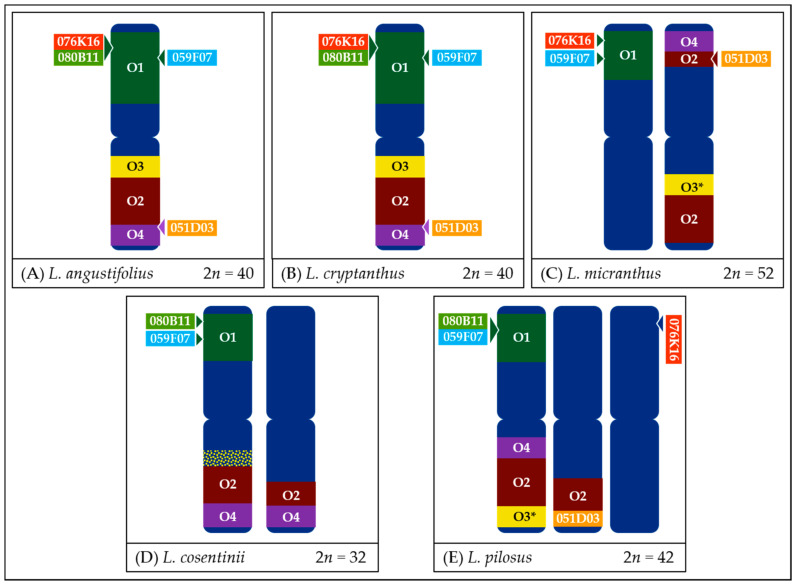
Schematic representation of probe mapping pattern, showing observed positions of particular oligonucleotide-based or BAC-based probes in *L. angustifolius* (**A**), *L. cryptanthus* (**B**), *L. micranthus* (**C**), *L. cosentinii* (**D**), and *L. pilosus* (**E**) chromosomes. In the case of probe O3 in *L. micranthus* and *L. pilosus*, beside two major loci, minor signals were also noticed. In *L. cosentinii*, the O3 probe hybridized to multiple loci. Chromosome schemes and probes length are not drawn to scale.

- *L. cryptanthus* (Figure 4 and Figure 8) is the only species tested with no significant structural differences detected compared to *L. angustifolius*, which supports the hypotheses of a close relationship between these two lupins.
- In the case of *L. micranthus* (Figure 5 and Figure 8), evolutionally the oldest among the studied species, the probes specific to the Lang06 chromosome landed on two different chromosomes, which may represent the pattern in the common ancestor of Old World lupins. During the course of evolution and speciation, the two genome fragments were translocated to one chromosome.
- In *L. cosentinii* (Figure 6 and Figure 8), hybridization of both O2 and O4 to two different chromosomes, as well as the highest number of probes (BACs and oligonucleotides) dispersed on multiple loci, might be the result of duplication and/or translocation of the arm B fragment of Lang06 chromosome (containing O1 and O2 probes) to Lcos06′ chromosome.
- Significant synteny changes detected in *L. pilosus* (Figure 7 and Figure 8) were probably the result of a series of rearrangements, including translocation, paracentric inversion, and/or non-allelic homologous recombination, leading to the separation of probes derived from Lang06 into three individual *L. pilosus* chromosomes.

## Figures and Tables

**Table 1:** General characteristics of the lupin species used in this study [20,21].

Group	Section	Species	Accession	Chromosome Number (2*n*)	Genome Size (pg/2C DNA)
Smooth-seeded	Angustifolius	*L. angustifolius* L.	cv. ‘Sonet’	40	1.89
		*L. cryptanthus* Shuttlew	96361	40	1.86
	Micranthus	*L. micranthus* Guss.	98552	52	0.98
Rough-seeded	Pilosus	*L. cosentinii* Guss.	98452	32	1.42
		*L. pilosus* Murr.	98653	42	1.36

**Table 2:** Characteristic of the designed oligonucleotides.

Oligo Library ID	Lang06 arm (A/B)	Number of Oligonucleotides in Pool	Covered Region in Template Sequence (bp)	Template Coverage	Average Density
O1	A	20,115	32,610–11,689,408	28.50%	0.58 oligo/kb
O2	B	19,926	29,400,911–40,902,159	28.19%	0.58 oligo/kb
O3	B	10,214	27,898,181–36,244,755	20.41%	0.82 oligo/kb
O4	B	8001	38,482,724–40,902,115	5.91%	0.30 oligo/kb

## Supplementary Materials

The following are available online at https://www.mdpi.com/2073-4425/11/12/1489/s1, Figure S1: FISH mapping of oligonucleotide probes in meiotic chromosomes of *L. angustifolius*, Table S1: Position of BAC clones in the pseudochromosome Lang06 of *L. angustifolius* cv. Tanjil mapped using BLAST in Geneious R9.1.8., Table S2: Alignment of BAC clone 076K16 to *L. albus* genome mapped using BLAST in White Lupin Genome Sequence Server 1.0.11. The detailed list of used oligonucleotide probes is available online at doi.org/10.5281/zenodo.4226537.
