# Supplementary material for: The Puzzling Fate of a Lupin Chromosome Revealed by Reciprocal Oligo-FISH and BAC-FISH Mapping
Source: Genes (Basel). 2020 Dec 10;11(12):1489. doi: 10.3390/genes11121489 (PMC7764521; doi:10.3390/genes11121489)
Supplement: Supplementary file 1 [file genes-11-01489-s001.zip › new_supplementary/Figure S1.docx]

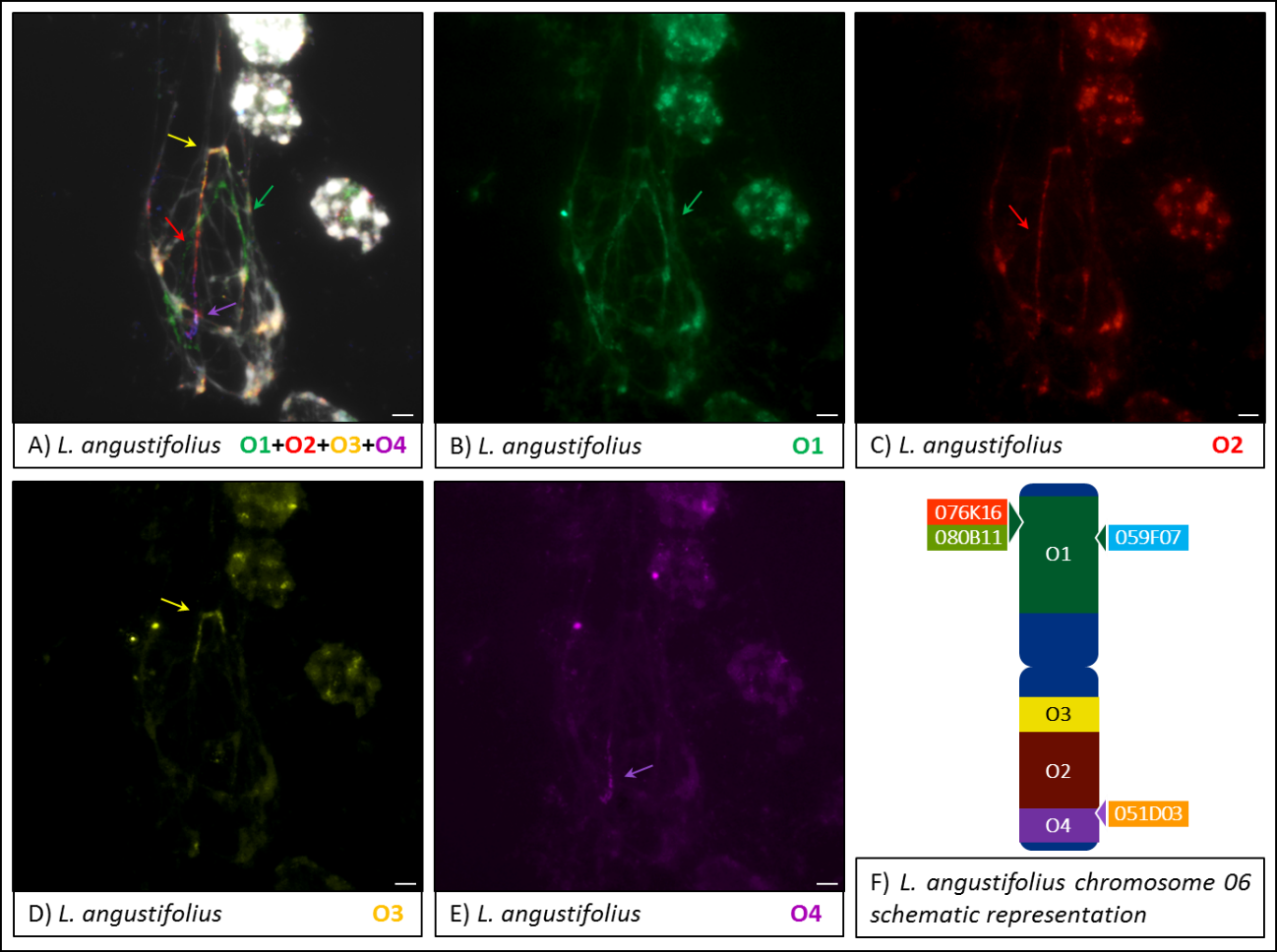


**Figure S1**. Fluorescence in situ hybridization (FISH) mapping of oligonucleotide probes in meiotic chromosomes of L. angustifolius. The positions of individual probes are marked by arrows. Probe colors are as follows: green (O1, Lang06 arm A), red (O2, Lang06 arm B), yellow (O3, pericentromeric region of Lang06 arm B) and purple (O4, telomere region of Lang06 arm B). Scale bar: 5 µm. The localizations of all probes were confirmed in meiotic chromosomes by two consecutive oligo-FISH reactions performed on the same slide. Composite image of oligo-FISH on meiotic sample is presented on Fig. S1A, whereas images of separate channels for individual probes on S1B – E. Chromosome Lang06 schematic representation (S1F), showing the positions of aligned particular oligonucleotide-based or BAC-based probes, was not drawn to scale.
