# Supplementary material for: The Puzzling Fate of a Lupin Chromosome Revealed by Reciprocal Oligo-FISH and BAC-FISH Mapping
Source: Genes (Basel). 2020 Dec 10;11(12):1489. doi: 10.3390/genes11121489 (PMC7764521; doi:10.3390/genes11121489)
Supplement: Supplementary file 1 [file genes-11-01489-s001.zip › new_supplementary/Table S1.docx]

**Table S1.** Positon of BAC clones in the pseudochromosome Lang06 of *L. angustifolius* cv. Tanjil [1] mapped using BLAST in Geneious R9.1.8. WBS – whole BAC sequence.

| **Lang** | **BAC clone**  **(length)** | **Identity** | **E-value** | **Query coverage** | **Sequence type** | **Genbank ID** | **Subject Start** | **Subject Stop** | **Query Start** | **Query Stop** |
| --- | --- | --- | --- | --- | --- | --- | --- | --- | --- | --- |
| 06 | 051D03 | 98.6% | 0 | 11.91% | WBS | MK650071.1 | 39,319,724 | 39,330,467 | 1 | 10,751 |
|  |  | 98.3% | 0 | 8.83% |  |  | 39,330,496 | 39,338,468 | 10,746 | 18,720 |
|  |  | 98.1% | 0 | 3.21% |  |  | 39,338,862 | 39,341,755 | 19,111 | 22,012 |
|  |  | 96.2% | 0 | 2.06% |  |  | 39,344,207 | 39,346,042 | 22,322 | 24,184 |
|  |  | 98.5% | 0 | 25.05% |  |  | 39,348,905 | 39,371,496 | 25,531 | 48,146 |
|  |  | 99.1% | 0 | 7.46% |  |  | 39,373,662 | 39,380,410 | 50,217 | 56,950 |
|  |  | 96.6% | 0 | 5.86% |  |  | 39,393,260 | 39,398,606 | 68,350 | 73,643 |
|  |  | 98.3% | 0 | 18.99% |  |  | 39,398,109 | 39,415,255 | 76,532 | 79,872 |
| 06 | 059F07 | 99.2% | 0 | 7.65% | WBS | KU678219.1 | 5,082,917 | 5,086,815 | 560 | 4459 |
|  |  | 99.5% | 0 | 7.04% |  |  | 5,087,945 | 5,091,535 | 5633 | 9223 |
|  |  | 97.6% | 0 | 8.29% |  |  | 5,091,576 | 5,095,864 | 9264 | 13,493 |
|  |  | 99.5% | 0 | 4.90% |  |  | 5,095,895 | 5,098,391 | 13,524 | 16,024 |
|  |  | 99.3% | 0 | 6.34% |  |  | 5,100,314 | 5,103,543 | 18,101 | 21,332 |
|  |  | 99.9% | 0 | 6.25% |  |  | 5,103,593 | 5,106,776 | 21,382 | 24,566 |
|  |  | 99.7% | 0 | 6.17% |  |  | 5,106,808 | 5,109,953 | 24,598 | 27,743 |
|  |  | 99.7% | 0 | 10.66% |  |  | 5,110,015 | 5,115,452 | 27,805 | 33,242 |
|  |  | 99.6% | 0 | 4.03% |  |  | 5,115,927 | 5,117,983 | 33,721 | 35,776 |
|  |  | 96.8% | 0 | 4.52% |  |  | 5,118,055 | 5,120,345 | 35,848 | 38,150 |
|  |  | 99.5% | 0 | 11.26% |  |  | 5,123,195 | 5,128,937 | 38,982 | 44,725 |
|  |  | 100% | 0 | 6.05% |  |  | 5,129,010 | 5,132,092 | 44,797 | 47,880 |
|  |  | 100% | 0 | 2.26% |  |  | 5,132,136 | 5,133,290 | 47,924 | 49,078 |
|  |  | 100% | 0 | 3.66% |  |  | 5,133,341 | 5,135,208 | 49,129 | 50,996 |
| 06 | 067H16 | 97.8% | 0 | 1.84% | WBS | HE804811.1 | 36,458,781 | 36,460,493 | 1 | 1713 |
|  |  | 97.2% | 0 | 1.70% |  |  | 36,457,140 | 36,458,734 | 1709 | 3290 |
|  |  | 95.4% | 0 | 6.30% |  |  | 36,448,309 | 36,454,200 | 6683 | 12,561 |
|  |  | 97.1% | 0 | 10.50% |  |  | 36,438,617 | 36,448,351 | 12,585 | 22,377 |
|  |  | 96.4% | 0 | 2.87% |  |  | 36,431,625 | 36,434,318 | 22,397 | 25,076 |
|  |  | 95.5% | 0 | 2.74% |  |  | 36,436,062 | 36,438,627 | 22,397 | 24,949 |
|  |  | 94.7% | 0 | 2.49% |  |  | 36,429,472 | 36,431,788 | 25,038 | 27,357 |
|  |  | 99.1% | 0 | 1.52% |  |  | 36,423,982 | 36,425,401 | 36,520 | 37,940 |
|  |  | 96.4% | 0 | 1.54% |  |  | 36,422,571 | 36,423,984 | 38,007 | 39,446 |
|  |  | 96.6% | 0 | 4.44% |  |  | 36,460,689 | 36,464,869 | 39,599 | 43,744 |
|  |  | 98.3% | 0 | 4.61% |  |  | 36,466,333 | 36,470,617 | 51,396 | 55,693 |
|  |  | 98.0% | 0 | 6.97% |  |  | 36,471,212 | 36,477,738 | 57,113 | 63,616 |
|  |  | 97.2% | 0 | 22.67% |  |  | 36,364,198 | 36,392,861 | 64,652 | 88,128 |
|  |  | 97.7% | 0 | 2.82% |  |  | 36,382,974 | 36,385,611 | 90,654 | 93,288 |
| 06 | 076K16 | 100% | 0 | 57.19% | WBS | MK650073.1 | 3,551,344 | 3,604,642 | 1 | 53,294 |
|  |  | 99.4% | 0 | 26.78% |  |  | 3,526,662 | 3,551,553 | 53,177 | 78,131 |
|  |  | 100% | 0 | 6.79% |  |  | 3,520,236 | 3,526,561 | 78,259 | 84,586 |
| 06 | 080B11 | 100% | 0 | 52.58% | WBS | HE804812.1 | 3 798 436 | 3 844 989 | 450 | 47,007 |
|  |  | 100% | 0 | 46.77% |  |  | 3 756 999 | 3 798 413 | 47 111 | 88,521 |
| 06 | 127N17 | 98.5% | 0 | 26.63% | WBS | KU678223.1 | 39,411,411 | 39,415,261 | 1 | 3822 |
|  |  | 96.7% | 0 | 32.92% |  |  | 39,406,627 | 39,411,365 | 3868 | 8592 |
|  |  | 98.9% | 0 | 39.79% |  |  | 39,400,864 | 39,406,577 | 8642 | 14,351 |

1. Zhou. G. et al. *Construction of an ultra-high density consensus genetic map. and enhancement of the physical map from genome sequencing in Lupinus angustifolius.* Theoretical and Applied Genetics. 2018. **131**(1): p. 209-223.
