# Supplementary material for: The Puzzling Fate of a Lupin Chromosome Revealed by Reciprocal Oligo-FISH and BAC-FISH Mapping
Source: Genes (Basel). 2020 Dec 10;11(12):1489. doi: 10.3390/genes11121489 (PMC7764521; doi:10.3390/genes11121489)
Supplement: Supplementary file 1 [file genes-11-01489-s001.zip › new_supplementary/Table S2.docx]

**Table S2.** Alignment of BAC clone 076K16 (GenBank ID: MK650073.1) to *L. albus* genome [1] mapped using BLAST in White Lupin Genome Sequence Server 1.0.11 (https://www.whitelupin.fr/sequenceServer.html). Bit score cut off = 200. sorted by pseudochromosome and query start position. Significant hits detected on chromosome Lalb05 (marked in green). Lalb09 (yellow) and Lalb16 (blue). Low quality hits marked in grey.

| query  acc.ver. | subject  acc.ver. | identity (%) | alignment length | mismatches | gap opens | query  start | query  end | subject  start | subject  end | e-value | bit score |
| --- | --- | --- | --- | --- | --- | --- | --- | --- | --- | --- | --- |
| MK650073.1 | Lalb_Chr03 | 84.122 | 296 | 39 | 5 | 41,066 | 41,356 | 2,675,554 | 2,675,846 | 6.89e-80 | 307.00 |
| MK650073.1 | Lalb_Chr03 | 90.000 | 200 | 15 | 3 | 41,709 | 41,904 | 2,681,766 | 2,681,964 | 2.57e-66 | 262.00 |
| MK650073.1 | Lalb_Chr03 | 91.558 | 154 | 9 | 1 | 42,318 | 42,471 | 2,681,052 | 2,681,201 | 2.75e-53 | 219.00 |
| MK650073.1 | Lalb_Chr03 | 95.522 | 134 | 5 | 1 | 44,502 | 44,634 | 2,682,078 | 2,682,211 | 4.08e-51 | 212.00 |
| MK650073.1 | Lalb_Chr03 | 86.800 | 500 | 38 | 2 | 61,309 | 61,784 | 2,681,052 | 2,681,547 | 2.40e-174 | 621.00 |
| MK650073.1 | Lalb_Chr03 | 87.812 | 361 | 24 | 4 | 61,784 | 62,129 | 2,681,609 | 2,681,964 | 7.35e-124 | 453.00 |
| MK650073.1 | Lalb_Chr05 | 89.412 | 170 | 5 | 1 | 1 | 157 | 2,393,090 | 2,392,921 | 1.25e-57 | 233.00 |
| MK650073.1 | Lalb_Chr05 | 84.589 | 863 | 49 | 3 | 156 | 939 | 2,392,864 | 2,392,007 | 0 | 1020.00 |
| MK650073.1 | Lalb_Chr05 | 89.302 | 215 | 20 | 2 | 1589 | 1800 | 2,391,399 | 2,391,185 | 3.34e-71 | 279.00 |
| MK650073.1 | Lalb_Chr05 | 87.594 | 266 | 14 | 3 | 2518 | 2778 | 2,390,817 | 2,390,566 | 4.96e-88 | 334.00 |
| MK650073.1 | Lalb_Chr05 | 96.781 | 1367 | 44 | 0 | 2838 | 4204 | 2,390,376 | 2,389,010 | 0 | 2268.00 |
| MK650073.1 | Lalb_Chr05 | 76.144 | 306 | 27 | 5 | 4544 | 4803 | 2,388,777 | 2,388,472 | 2.41e-60 | 242.00 |
| MK650073.1 | Lalb_Chr05 | 75.062 | 401 | 63 | 14 | 7823 | 8218 | 2,387,313 | 2,386,945 | 2.41e-60 | 242.00 |
| MK650073.1 | Lalb_Chr05 | 70.316 | 1014 | 69 | 22 | 8482 | 9350 | 2,386,849 | 2,385,923 | 2.10e-162 | 581.00 |
| MK650073.1 | Lalb_Chr05 | 80.585 | 376 | 34 | 6 | 13,857 | 14,221 | 2,384,743 | 2,384,396 | 4.35e-95 | 358.00 |
| MK650073.1 | Lalb_Chr05 | 82.992 | 635 | 27 | 8 | 14,809 | 15,394 | 2,383,968 | 2,383,366 | 0 | 695.00 |
| MK650073.1 | Lalb_Chr05 | 92.143 | 700 | 38 | 4 | 15,514 | 16,203 | 2,383,291 | 2,382,599 | 0 | 1012.00 |
| MK650073.1 | Lalb_Chr05 | 95.122 | 164 | 8 | 0 | 16,198 | 16,361 | 2,382,532 | 2,382,369 | 8.98e-66 | 260.00 |
| MK650073.1 | Lalb_Chr05 | 97.674 | 172 | 4 | 0 | 16,515 | 16,686 | 2,382,023 | 2,381,852 | 1.52e-75 | 293.00 |
| MK650073.1 | Lalb_Chr05 | 92.191 | 397 | 12 | 3 | 16,737 | 17,129 | 2,381,758 | 2,381,377 | 7.34e-162 | 580.00 |
| MK650073.1 | Lalb_Chr05 | 91.255 | 263 | 17 | 1 | 17,258 | 17,514 | 2,381,355 | 2,381,093 | 1.97e-99 | 372.00 |
| MK650073.1 | Lalb_Chr05 | 81.156 | 467 | 25 | 8 | 17,534 | 17,946 | 2,380,732 | 2,380,275 | 1.16e-127 | 466.00 |
| MK650073.1 | Lalb_Chr05 | 81.197 | 234 | 23 | 4 | 18,171 | 18,394 | 2,379,964 | 2,379,742 | 6.46e-55 | 224.00 |
| MK650073.1 | Lalb_Chr05 | 94.652 | 187 | 9 | 1 | 18,392 | 18,577 | 2,379,676 | 2,379,490 | 1.85e-74 | 289.00 |
| MK650073.1 | Lalb_Chr05 | 65.324 | 571 | 95 | 10 | 19,297 | 19,765 | 2,379,159 | 2,378,590 | 7.87e-54 | 221.00 |
| MK650073.1 | Lalb_Chr05 | 79.116 | 249 | 22 | 5 | 20,163 | 20,382 | 2,378,252 | 2,378,005 | 2.75e-53 | 219.00 |
| MK650073.1 | Lalb_Chr05 | 85.921 | 760 | 74 | 6 | 20,392 | 21,150 | 2,377,934 | 2,377,207 | 0 | 892.00 |
| MK650073.1 | Lalb_Chr05 | 88.804 | 652 | 50 | 8 | 21,149 | 21,784 | 2,377,132 | 2,376,488 | 0 | 832.00 |
| MK650073.1 | Lalb_Chr05 | 96.098 | 205 | 8 | 0 | 21,784 | 21,988 | 2,376,423 | 2,376,219 | 4.96e-88 | 334.00 |
| MK650073.1 | Lalb_Chr05 | 84.314 | 867 | 35 | 7 | 21,996 | 22,771 | 2,376,123 | 2,375,267 | 0 | 1011.00 |
| MK650073.1 | Lalb_Chr05 | 88.725 | 204 | 21 | 1 | 22,901 | 23,104 | 2,375,272 | 2,375,071 | 2.57e-66 | 262.00 |
| MK650073.1 | Lalb_Chr05 | 80.741 | 270 | 32 | 6 | 23,117 | 23,376 | 2,374,935 | 2,374,676 | 6.90e-61 | 244.00 |
| MK650073.1 | Lalb_Chr05 | 91.283 | 608 | 37 | 5 | 24,354 | 24,955 | 2,374,229 | 2,373,632 | 0 | 850.00 |
| MK650073.1 | Lalb_Chr05 | 76.633 | 398 | 93 | 0 | 24,504 | 24,901 | 16,325,580 | 16,325,183 | 3.57e-77 | 298.00 |
| MK650073.1 | Lalb_Chr05 | 79.087 | 416 | 80 | 4 | 25,300 | 25,712 | 16,325,098 | 16,324,687 | 7.86e-92 | 347.00 |
| MK650073.1 | Lalb_Chr05 | 88.016 | 1961 | 102 | 10 | 25,309 | 27,147 | 2,373,616 | 2,371,667 | 0 | 2553.00 |
| MK650073.1 | Lalb_Chr05 | 80 | 375 | 67 | 4 | 26,227 | 26,593 | 16,323,057 | 16,322,683 | 7.36e-86 | 327.00 |
| MK650073.1 | Lalb_Chr05 | 68.148 | 405 | 50 | 3 | 26,766 | 27,091 | 16,322,109 | 16,321,705 | 1.73e-49 | 206.00 |
| MK650073.1 | Lalb_Chr05 | 86.188 | 362 | 24 | 6 | 27,614 | 27,963 | 2,371,474 | 2,371,127 | 3.57e-115 | 425.00 |
| MK650073.1 | Lalb_Chr05 | 82.68 | 612 | 63 | 8 | 27,964 | 28,549 | 2,371,006 | 2,370,412 | 1.61e-176 | 628.00 |
| MK650073.1 | Lalb_Chr05 | 76.157 | 432 | 37 | 8 | 30,914 | 31,324 | 2,368,479 | 2,368,093 | 4.07e-89 | 338.00 |
| MK650073.1 | Lalb_Chr05 | 77.778 | 1044 | 117 | 20 | 31,653 | 32,608 | 2,366,259 | 2,365,243 | 0 | 850.00 |
| MK650073.1 | Lalb_Chr05 | 89.038 | 520 | 32 | 6 | 32,652 | 33,152 | 4,315,840 | 4,315,327 | 0 | 677.00 |
| MK650073.1 | Lalb_Chr05 | 82.558 | 258 | 29 | 4 | 33,160 | 33,411 | 4,308,334 | 4,308,087 | 3.13e-65 | 259.00 |
| MK650073.1 | Lalb_Chr05 | 80 | 235 | 22 | 7 | 33,416 | 33,647 | 4,305,916 | 4,305,704 | 2.11e-48 | 203.00 |
| MK650073.1 | Lalb_Chr05 | 77.308 | 639 | 61 | 12 | 33,644 | 34,208 | 3,427,873 | 3,428,501 | 6.03e-144 | 520.00 |
| MK650073.1 | Lalb_Chr05 | 87.636 | 922 | 85 | 5 | 34,506 | 35,424 | 3,429,184 | 3,430,079 | 0 | 1153.00 |
| MK650073.1 | Lalb_Chr05 | 86.081 | 273 | 28 | 3 | 35,497 | 35,767 | 3,430,140 | 3,430,404 | 1.33e-82 | 316.00 |
| MK650073.1 | Lalb_Chr05 | 78.406 | 389 | 40 | 8 | 35,764 | 36,112 | 3,430,504 | 3,430,888 | 7.36e-86 | 327.00 |
| MK650073.1 | Lalb_Chr05 | 78.537 | 410 | 34 | 4 | 36,192 | 36,548 | 4,305,718 | 4,305,310 | 5.65e-100 | 374.00 |
| MK650073.1 | Lalb_Chr05 | 88.372 | 387 | 22 | 5 | 36,741 | 37,105 | 4,305,195 | 4,304,810 | 8.38e-136 | 493.00 |
| MK650073.1 | Lalb_Chr05 | 87.245 | 196 | 24 | 1 | 37,211 | 37,405 | 1,388,144 | 1,387,949 | 1.02e-58 | 237.00 |
| MK650073.1 | Lalb_Chr05 | 78.317 | 309 | 37 | 4 | 37,428 | 37,735 | 2,360,736 | 2,360,457 | 7.37e-67 | 264.00 |
| MK650073.1 | Lalb_Chr05 | 82.667 | 225 | 36 | 2 | 38,714 | 38,938 | 4,309,706 | 4,309,485 | 6.46e-55 | 224.00 |
| MK650073.1 | Lalb_Chr05 | 82.211 | 950 | 94 | 12 | 39,115 | 40,052 | 7,368,881 | 7,369,767 | 0 | 966.00 |
| MK650073.1 | Lalb_Chr05 | 91.739 | 230 | 16 | 2 | 42,589 | 42,815 | 2,358,821 | 2,358,592 | 8.97e-85 | 324.00 |
| MK650073.1 | Lalb_Chr05 | 84.718 | 301 | 39 | 3 | 43,191 | 43,490 | 1,387,649 | 1,387,355 | 2.11e-86 | 329.00 |
| MK650073.1 | Lalb_Chr05 | 92.287 | 363 | 21 | 3 | 44,662 | 45,018 | 4,303,393 | 4,303,032 | 1.73e-144 | 522.00 |
| MK650073.1 | Lalb_Chr05 | 88.824 | 340 | 29 | 3 | 45,892 | 46,223 | 2,359,395 | 2,359,057 | 5.65e-119 | 437.00 |
| MK650073.1 | Lalb_Chr05 | 88.529 | 401 | 30 | 1 | 46,536 | 46,936 | 2,358,976 | 2,358,592 | 1.42e-145 | 526.00 |
| MK650073.1 | Lalb_Chr05 | 82.09 | 402 | 46 | 9 | 52,706 | 53,095 | 3,428,114 | 3,428,501 | 1.09e-102 | 383.00 |
| MK650073.1 | Lalb_Chr05 | 84.517 | 704 | 92 | 5 | 53,090 | 53,776 | 3,428,653 | 3,429,356 | 0 | 771.00 |
| MK650073.1 | Lalb_Chr05 | 79.96 | 494 | 42 | 8 | 53,806 | 54,278 | 4,305,916 | 4,305,459 | 4.95e-126 | 461.00 |
| MK650073.1 | Lalb_Chr05 | 88.58 | 324 | 33 | 4 | 54,457 | 54,777 | 4,305,165 | 4,304,843 | 1.17e-108 | 403.00 |
| MK650073.1 | Lalb_Chr05 | 92.818 | 362 | 23 | 2 | 55,260 | 55,618 | 4,304,762 | 4,304,401 | 1.16e-146 | 529.00 |
| MK650073.1 | Lalb_Chr05 | 92.347 | 196 | 15 | 0 | 55,615 | 55,810 | 4,304,314 | 4,304,119 | 2.25e-73 | 286.00 |
| MK650073.1 | Lalb_Chr05 | 92.241 | 232 | 17 | 1 | 56,062 | 56,292 | 4,303,300 | 4,303,069 | 4.96e-88 | 334.00 |
| MK650073.1 | Lalb_Chr05 | 69.196 | 448 | 64 | 7 | 56,841 | 57,229 | 1,388,431 | 1,387,999 | 7.87e-54 | 221.00 |
| MK650073.1 | Lalb_Chr05 | 79.47 | 302 | 32 | 5 | 57,270 | 57,571 | 2,360,736 | 2,360,465 | 1.73e-68 | 269.00 |
| MK650073.1 | Lalb_Chr05 | 89.792 | 529 | 32 | 5 | 57,718 | 58,245 | 2,360,122 | 2,359,615 | 0 | 708.00 |
| MK650073.1 | Lalb_Chr05 | 87.316 | 339 | 27 | 1 | 58,401 | 58,739 | 2,358,976 | 2,358,654 | 2.93e-116 | 428.00 |
| MK650073.1 | Lalb_Chr05 | 79.89 | 547 | 51 | 7 | 58,871 | 59,369 | 2,358,371 | 2,357,836 | 8.94e-142 | 513.00 |
| MK650073.1 | Lalb_Chr05 | 78.179 | 637 | 95 | 15 | 58,922 | 59,547 | 2,358,279 | 2,357,676 | 2.40e-136 | 495.00 |
| MK650073.1 | Lalb_Chr05 | 84.08 | 647 | 76 | 8 | 60,225 | 60,868 | 7,369,145 | 7,369,767 | 0 | 691.00 |
| MK650073.1 | Lalb_Chr05 | 79.475 | 419 | 54 | 5 | 62,835 | 63,223 | 2,358,821 | 2,358,405 | 5.65e-100 | 374.00 |
| MK650073.1 | Lalb_Chr05 | 76.923 | 494 | 92 | 8 | 63,223 | 63,709 | 1,388,911 | 1,388,433 | 3.57e-96 | 361.00 |
| MK650073.1 | Lalb_Chr05 | 68.254 | 504 | 46 | 12 | 65,374 | 65,779 | 2,370,265 | 2,369,778 | 1.02e-58 | 237.00 |
| MK650073.1 | Lalb_Chr05 | 85.648 | 216 | 26 | 1 | 69,972 | 70,182 | 7,369,214 | 7,368,999 | 1.62e-62 | 250.00 |
| MK650073.1 | Lalb_Chr05 | 87.356 | 174 | 16 | 1 | 70,351 | 70,524 | 4,315,747 | 4,315,914 | 3.35e-52 | 215.00 |
| MK650073.1 | Lalb_Chr05 | 81.692 | 650 | 69 | 12 | 70,644 | 71,256 | 2,365,453 | 2,366,089 | 5.63e-176 | 627.00 |
| MK650073.1 | Lalb_Chr08 | 88.055 | 293 | 20 | 2 | 45,541 | 45,818 | 15,384,984 | 15,385,276 | 1.62e-100 | 376.00 |
| MK650073.1 | Lalb_Chr08 | 88.055 | 293 | 20 | 2 | 47,159 | 47,436 | 15,384,984 | 15,385,276 | 1.62e-100 | 376.00 |
| MK650073.1 | Lalb_Chr09 | 92.381 | 1365 | 104 | 0 | 2840 | 4204 | 3,127,869 | 3,126,505 | 0.0 | 1994.00 |
| MK650073.1 | Lalb_Chr09 | 77.907 | 344 | 50 | 5 | 4207 | 4546 | 3,126,408 | 3,126,087 | 3.34e-71 | 279.00 |
| MK650073.1 | Lalb_Chr09 | 81.671 | 742 | 71 | 8 | 4969 | 5678 | 3,125,320 | 3,124,612 | 0.0 | 747.00 |
| MK650073.1 | Lalb_Chr09 | 72.283 | 552 | 98 | 6 | 5016 | 5545 | 14,247,774 | 14,247,256 | 2.11e-86 | 329.00 |
| MK650073.1 | Lalb_Chr09 | 69.335 | 812 | 108 | 21 | 14,607 | 15,334 | 3,114,987 | 3,114,233 | 5.65e-100 | 374.00 |
| MK650073.1 | Lalb_Chr09 | 86.971 | 591 | 67 | 6 | 15,594 | 16,177 | 3,114,174 | 3,113,587 | 0.0 | 700.00 |
| MK650073.1 | Lalb_Chr09 | 74.558 | 566 | 118 | 10 | 15,614 | 16,177 | 14,193,605 | 14,193,064 | 6.45e-93 | 351.00 |
| MK650073.1 | Lalb_Chr09 | 76.970 | 330 | 54 | 6 | 15,846 | 16,175 | 14,221,290 | 14,221,597 | 1.98e-61 | 246.00 |
| MK650073.1 | Lalb_Chr09 | 94.074 | 135 | 8 | 0 | 16,517 | 16,651 | 3,112,927 | 3,112,793 | 4.97e-50 | 208.00 |
| MK650073.1 | Lalb_Chr09 | 85.530 | 774 | 83 | 7 | 16,744 | 17,514 | 3,112,771 | 3,112,024 | 0.0 | 886.00 |
| MK650073.1 | Lalb_Chr09 | 85.199 | 277 | 31 | 3 | 17,534 | 17,800 | 3,111,741 | 3,111,465 | 5.66e-81 | 311.00 |
| MK650073.1 | Lalb_Chr09 | 67.597 | 1003 | 150 | 30 | 31,653 | 32,582 | 3,104,601 | 3,103,701 | 8.39e-98 | 367.00 |
| MK650073.1 | Lalb_Chr09 | 80.294 | 340 | 47 | 6 | 32,603 | 32,933 | 4,598,973 | 4,598,645 | 2.93e-78 | 302.00 |
| MK650073.1 | Lalb_Chr09 | 75.000 | 352 | 36 | 4 | 36,204 | 36,504 | 4,573,576 | 4,573,926 | 6.05e-68 | 268.00 |
| MK650073.1 | Lalb_Chr09 | 78.000 | 550 | 84 | 11 | 36,573 | 37,085 | 4,574,185 | 4,574,734 | 8.38e-117 | 430.00 |
| MK650073.1 | Lalb_Chr09 | 77.431 | 545 | 87 | 11 | 38,185 | 38,707 | 4,588,805 | 4,588,275 | 2.25e-111 | 412.00 |
| MK650073.1 | Lalb_Chr09 | 77.920 | 548 | 60 | 7 | 39,109 | 39,600 | 7,622,611 | 7,623,153 | 1.16e-127 | 466.00 |
| MK650073.1 | Lalb_Chr09 | 78.684 | 380 | 56 | 6 | 39,586 | 39,950 | 7,623,555 | 7,623,924 | 1.33e-82 | 316.00 |
| MK650073.1 | Lalb_Chr09 | 81.590 | 239 | 39 | 3 | 43,180 | 43,415 | 13,535,237 | 13,535,001 | 6.46e-55 | 224.00 |
| MK650073.1 | Lalb_Chr09 | 85.165 | 364 | 43 | 6 | 44,662 | 45,018 | 4,576,101 | 4,576,460 | 1.73e-106 | 396.00 |
| MK650073.1 | Lalb_Chr09 | 75.794 | 504 | 62 | 11 | 53,807 | 54,278 | 4,573,348 | 4,573,823 | 1.02e-96 | 363.00 |
| MK650073.1 | Lalb_Chr09 | 79.038 | 520 | 71 | 12 | 54,457 | 54,942 | 4,574,396 | 4,574,911 | 1.02e-115 | 426.00 |
| MK650073.1 | Lalb_Chr09 | 85.308 | 422 | 55 | 6 | 55,201 | 55,618 | 4,575,020 | 4,575,438 | 4.95e-126 | 461.00 |
| MK650073.1 | Lalb_Chr09 | 87.166 | 187 | 22 | 2 | 55,615 | 55,801 | 4,575,525 | 4,575,709 | 2.26e-54 | 223.00 |
| MK650073.1 | Lalb_Chr09 | 87.391 | 230 | 27 | 2 | 56,065 | 56,292 | 4,576,195 | 4,576,424 | 1.17e-70 | 277.00 |
| MK650073.1 | Lalb_Chr09 | 83.000 | 200 | 26 | 3 | 60,224 | 60,416 | 7,622,955 | 7,623,153 | 7.37e-48 | 201.00 |
| MK650073.1 | Lalb_Chr09 | 78.919 | 370 | 53 | 6 | 60,412 | 60,766 | 7,623,565 | 7,623,924 | 5.66e-81 | 311.00 |
| MK650073.1 | Lalb_Chr09 | 73.158 | 380 | 40 | 12 | 70,927 | 71,256 | 3,104,050 | 3,104,417 | 5.31e-56 | 228.00 |
| MK650073.1 | Lalb_Chr16 | 82.589 | 672 | 59 | 5 | 321 | 943 | 2,370,254 | 2,369,592 | 0.0 | 715.00 |
| MK650073.1 | Lalb_Chr16 | 79.848 | 263 | 46 | 5 | 1527 | 1783 | 2,368,298 | 2,368,037 | 2.75e-53 | 219.00 |
| MK650073.1 | Lalb_Chr16 | 91.667 | 216 | 16 | 2 | 3725 | 3940 | 2,367,572 | 2,367,359 | 2.93e-78 | 302.00 |
| MK650073.1 | Lalb_Chr16 | 71.253 | 487 | 62 | 10 | 4068 | 4550 | 2,367,325 | 2,366,913 | 1.42e-69 | 273.00 |
| MK650073.1 | Lalb_Chr16 | 73.775 | 408 | 51 | 7 | 4544 | 4906 | 2,366,863 | 2,366,467 | 1.42e-69 | 273.00 |
| MK650073.1 | Lalb_Chr16 | 82.781 | 784 | 74 | 8 | 4923 | 5678 | 2,366,312 | 2,365,562 | 0.0 | 825.00 |
| MK650073.1 | Lalb_Chr16 | 82.684 | 231 | 29 | 1 | 8371 | 8601 | 2,356,840 | 2,356,621 | 2.41e-60 | 242.00 |
| MK650073.1 | Lalb_Chr16 | 65.828 | 676 | 102 | 18 | 8679 | 9236 | 2,356,365 | 2,355,701 | 1.17e-51 | 214.00 |
| MK650073.1 | Lalb_Chr16 | 83.176 | 743 | 89 | 13 | 24,277 | 25,003 | 2,343,278 | 2,342,556 | 0.0 | 751.00 |
| MK650073.1 | Lalb_Chr16 | 78.219 | 2066 | 245 | 22 | 25,207 | 27,091 | 2,342,542 | 2,340,501 | 0.0 | 1783.00 |
| MK650073.1 | Lalb_Chr16 | 89.189 | 296 | 24 | 2 | 37,111 | 37,405 | 1,329,149 | 1,328,861 | 2.57e-104 | 389.00 |
| MK650073.1 | Lalb_Chr16 | 88.142 | 565 | 43 | 7 | 42,998 | 43,555 | 1,328,728 | 1,328,181 | 0.0 | 708.00 |
| MK650073.1 | Lalb_Chr16 | 71.819 | 951 | 169 | 20 | 46,056 | 46,928 | 2,327,652 | 2,326,723 | 1.33e-139 | 506.00 |
| MK650073.1 | Lalb_Chr16 | 92.754 | 138 | 10 | 0 | 56,615 | 56,752 | 1,330,018 | 1,329,881 | 6.05e-49 | 205.00 |
| MK650073.1 | Lalb_Chr16 | 86.869 | 396 | 34 | 6 | 56,837 | 57,229 | 1,329,291 | 1,328,911 | 9.56e-129 | 470.00 |
| MK650073.1 | Lalb_Chr16 | 84.358 | 537 | 60 | 10 | 57,720 | 58,244 | 2,328,488 | 2,327,964 | 4.63e-158 | 567.00 |
| MK650073.1 | Lalb_Chr16 | 81.281 | 406 | 65 | 4 | 58,331 | 58,725 | 2,327,203 | 2,326,798 | 3.81e-102 | 381.00 |
| MK650073.1 | Lalb_Chr16 | 81.201 | 383 | 65 | 3 | 58,928 | 59,305 | 2,326,419 | 2,326,039 | 1.25e-95 | 360.00 |
| MK650073.1 | Lalb_Chr16 | 79.644 | 506 | 70 | 7 | 63,221 | 63,719 | 1,329,758 | 1,329,279 | 1.09e-121 | 446.00 |
| MK650073.1 | Lalb_Chr16 | 76.589 | 299 | 51 | 5 | 63,272 | 63,563 | 10,427,923 | 10,427,637 | 2.75e-53 | 219.00 |
| MK650073.1 | Lalb_Chr18 | 80.237 | 253 | 42 | 3 | 44,033 | 44,277 | 15,084,080 | 15,084,332 | 6.46e-55 | 224.00 |
| MK650073.1 | Lalb_Chr18 | 78.457 | 376 | 66 | 6 | 55,249 | 55,613 | 19,413,510 | 19,413,139 | 1.02e-77 | 300.00 |
| MK650073.1 | Lalb_Chr18 | 79.300 | 343 | 48 | 6 | 64,160 | 64,482 | 15,084,083 | 15,084,422 | 1.52e-75 | 293.00 |
| MK650073.1 | Lalb_Chr18 | 78.767 | 292 | 39 | 10 | 67,923 | 68,198 | 15,082,987 | 15,083,271 | 2.26e-54 | 223.00 |
| MK650073.1 | Lalb_Chr18 | 81.048 | 248 | 34 | 8 | 67,959 | 68,198 | 15,080,363 | 15,080,605 | 4.08e-51 | 212.00 |
| MK650073.1 | Lalb_Chr19 | 87.560 | 209 | 23 | 1 | 42,263 | 42,471 | 15,466,971 | 15,466,766 | 3.13e-65 | 259.00 |
| MK650073.1 | Lalb_Chr19 | 86.447 | 273 | 34 | 1 | 61,254 | 61,526 | 15,466,971 | 15,466,702 | 8.97e-85 | 324.00 |
| MK650073.1 | Lalb_Chr20 | 84.615 | 273 | 21 | 6 | 43,764 | 44,022 | 2,358,394 | 2,358,659 | 4.35e-76 | 295.00 |
| MK650073.1 | Lalb_Chr20 | 80.690 | 435 | 36 | 11 | 63,715 | 64,123 | 2,358,231 | 2,358,643 | 1.42e-107 | 399.00 |
| MK650073.1 | Lalb_Chr25 | 73.515 | 404 | 47 | 16 | 63,749 | 64,113 | 14,802,917 | 14,803,299 | 5.31e-56 | 228.00 |

1. Hufnagel. B. et al.. *High-quality genome sequence of white lupin provides insight into soil exploration and seed quality.* Nature Communications. 2020. **11**(1): p. 1-12.
